# Effect of *Porphyromonas gingivalis* Infection on PGC‐1α in Skeletal Muscle After Endurance Training In Vivo

**DOI:** 10.1002/cre2.70415

**Published:** 2026-07-16

**Authors:** Kairi Hayashi, Yasuo Takeuchi, Hiroaki Kobayashi, Gen Tanabe, Hiroshi Churei, Toshiaki Ueno, Kenji Fueki

**Affiliations:** ^1^ Department of Masticatory Function and Health Science Graduate School of Medical and Dental Sciences, Institute of Science Tokyo Tokyo Japan; ^2^ Division of Sports Dentistry Sports Science Organization, Institute of Science Tokyo Tokyo Japan; ^3^ Department of Lifetime Oral Health Care Science Graduate School of Medical and Dental Sciences, Institute of Science Tokyo Tokyo Japan; ^4^ Department of Periodontology Graduate School of Medical and Dental Sciences, Institute of Science Tokyo Tokyo Japan; ^5^ Department of Sports Dentistry Meikai University School of Dentistry Sakado Japan

**Keywords:** endurance training, *P. gingivalis*, PGC‐1α, skeletal muscle

## Abstract

**Objectives:**

It is well known that *Porphyromonas gingivalis*, a major pathogen in periodontitis, causes systemic diseases. This study investigated the expression of PGC‐1α (the most important factor in muscle mitochondria synthesis) following endurance training to clarify the relationship between *P. gingivalis* infection and muscle endurance capacity.

**Materials and Methods:**

Twenty 8‐week‐old Wistar rats were randomly divided into two groups. One group was intraperitoneally administered sonicated *P. gingivalis* (*P. g*‐group), and the other group was administered saline (Control‐group). Three weeks after administration, gastrocnemius muscles and blood from both groups were collected 24 h after treadmill exercise. The expressions of PGC‐1α and TNF‐α in gastrocnemius muscle were measured, along with serum antibody titers for IgG, in response to *P. gingivalis*.

**Results:**

Serum IgG antibody titers and TNF‐α expression in skeletal muscle were significantly higher in the *P. g*‐group than in the Control‐group. PGC‐1α expression was significantly lower in the *P. g*‐group than in the Control‐group. A correlation was observed between PGC‐1α and TNF‐α expression.

**Conclusion:**

Our result suggests that the increase in endurance following training may be suppressed by *P. gingivalis* infection.

## 1 Introduction

It is well known that *Porphyromonas gingivalis* (*P. gingivalis*), a major pathogen in periodontal disease, is related not only to the oral environment but also to many systemic diseases. Previous studies have linked *P. gingivalis* to diabetes, cardiovascular disease, and low birth weight (Baeza et al. 2020; Naderi and Merchant 2020; Preshaw et al. 2012; Sanz et al. 2020; Teshome and Yitayeh 2016; Udagawa et al. 2018).

*Porphyromonas gingivalis* can invade blood vessels by passing through the endothelial cells of the periodontal pocket. Its bacterial toxins and components, such as lipopolysaccharide (LPS, an endotoxin), travel through the bloodstream (Dorn et al. 2002). This upregulates inflammatory cytokines such as interleukin‐6 (IL‐6) and tumor necrosis factor‐α (TNF‐α) throughout the body (Cardoso et al. 2018; Naruishi and Nagata 2018).

Additionally, *P. gingivalis* affects skeletal muscle. Watanabe et al. (2021) reported fat infiltration and lower glucose uptake in the skeletal muscle of *P. gingivalis‐*administered mice. Our previous study found that infection with might disrupt muscle satellite cell expression after in vivo damage (Hayashi et al. 2022). *P. gingivalis*

In this study, we focused on the energy metabolism of skeletal muscle. Energy metabolism is primarily related to mitochondrial function. Adenosine triphosphate (ATP) is an important molecule related to energy metabolism, synthesized through the citric acid cycle and the electron transport chain in the mitochondrial matrix and membrane (Chandel 2021). ATP is consumed during muscular activity, and muscle endurance is improved with an increased number of mitochondria or enhanced mitochondrial function.

Athletes, sports enthusiasts, and health‐conscious individuals regularly engage in muscle endurance training to enhance their muscle endurance. Endurance training results in increased mitochondrial expression and function through the expression of p38MK and peroxisome proliferator‐activated receptor gamma coactivator 1‐alpha (PGC‐1α) (Olesen et al. 2010). Muscle endurance training affects athletic performance and overall health. Improved muscle endurance is associated with basic physical strength. Kodama (2009) reported that basic physical strength is related to the mortality rate. Therefore, enhancing muscle endurance through exercise is crucial for overall health improvement.

Although *P. gingivalis* is a natural part of the oral microbiome, this microorganism is regarded as a “keystone” of oral microbial dysbiosis. Periodontitis results from a shift in the microbial community, leading to an increased accumulation of harmful bacteria (Cheng et al. 2021). *P. gingivalis* is detected not only in patients with periodontitis. For example, Carrouel et al. (2016) reported that *P. gingivalis* was detected in clinically healthy adolescents and young adults without any periodontal lesions (Carrouel et al. 2016). Therefore, understanding the effect of *P. gingivalis* infection on muscle endurance is important for improving the health of not only middle‐aged and elderly individuals prone to developing periodontal disease but also of young ones (including athletes) who do not exhibit any symptoms of the disease yet.

Some previous studies have reported that the expression of PGC‐1α, one of the most important factors in mitochondrial expression and function, is suppressed by the chronic increase of inflammatory cytokines such as TNF‐α (Palomer et al. 2009). *P. gingivalis* infection causes a chronic increase in inflammatory cytokines. Therefore, we hypothesized that infection with *P. gingivalis* may suppress the ability to improve muscle endurance after training. In our previous study (Hayashi et al. 2022), we reported lower endurance test scores in rats that had been previously infected with *P. gingivalis* compared to controls. To further investigate this, we examined the relationship between *P. gingivalis* infection and PGC‐1α expression after endurance training in vivo, aiming to clarify whether muscle endurance was affected as a consequence.

## 2 Material and Methods

### 2.1 Animals

**Figure 1:**
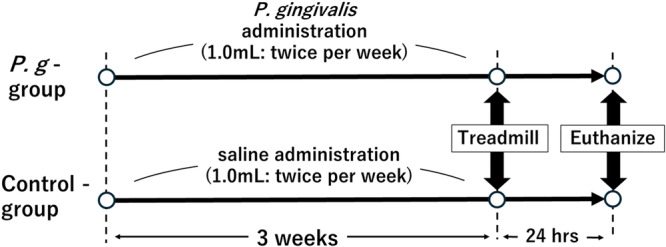
Schema of study protocol. The rats were randomly divided into two groups. Ten rats were intraperitoneally administered sonicated *P. gingivalis* (*P. g*‐group), and 10 were administered saline instead (Control group). Three weeks after administration, both groups of rats underwent treadmill exercise and were euthanized 24 h after the exercise session.

Twenty 8‐week‐old male Wistar rats were used in this study (Sankyo Labo Service Corporation Inc., Tokyo, Japan). The rats were randomly divided into two groups. Ten rats were intraperitoneally administered sonicated *P. gingivalis* (*P. g*‐group), and the remaining 10 were administered saline instead (Control‐group). Three weeks after administration, both groups were subjected to treadmill exercise and euthanized after 24 h (Figure 1). All experimental subjects were housed under the same conditions and provided with food and water *ad libitum*. To evaluate the effect of the *P. gingivalis* administration on dietary intake, body weight was measured daily using a digital scale (GF‐8K; A&D Co. Ltd., Tokyo, Japan).

All animal experiments were approved by the Institutional Animal Care and Use Committee of the Institute of Science Tokyo (A2022‐094C2), and efforts were made to minimize pain or discomfort in accordance with the Guidelines of the National Institute of Health. This study was conducted in accordance with the ARRIVE 2.0 guidelines for reporting animal research.

### 2.2 Administration of *Porphyromonas gingivalis*

*Porphyromonas gingivalis* (ATCC 33277) was anaerobically inoculated into a brain‐heart infusion broth medium supplemented with 5 mg/L of hemin and 50 μg/L of vitamin K1, and cultured for 2 days at 35°C. After sonication, bacterial cells were counted using a bacterial counting chamber. The concentration was adjusted to 10^9^ cells/mL, and the solution was centrifuged at 8000 × *g* for 10 min at 4°C. The pellet was resuspended in physiological saline solution. The rats in the *P. g*‐group were intraperitoneally administered with 1.0 mL of saline containing sonicated *P. gingivalis* twice per week during 3 weeks. The rats in the Control‐group were administered the same amount of saline as the *P. g*‐group.

### 2.3 Endurance Training

After 3 weeks of sonicated *P. gingivalis* administration, all rats were subjected to exercise on a treadmill (MK‐680; Wakenyaku Co. Ltd., Kyoto, Japan) at a speed of 22 m/min for 30 min.

### 2.4 Serum Antibody Titers for IgG in Response to *Porphyromonas gingivalis*

Twenty‐four hours after the exercise session, all rats were euthanized using an overdose of carbon dioxide. Blood samples were collected by cardiac puncture immediately after euthanasia. The blood was allowed to clot at room temperature, and the serum was separated by centrifugation at 1500 *g* for 15 min. The serum was collected and stored at −80°C until analysis. Serum antibody titers for IgG were measured using an enzyme‐linked immunosorbent assay, as previously described (Ohtsu et al. 2019; Wang et al. 2006). First, 96‐well microplates (GDMP‐96F; AS ONE CORPORATION, Osaka, Japan) were coated with sonicated *P. gingivalis* ATCC 33277 at 10 µg/mL in a carbonate buffer, and a serially diluted reference positive control serum (2^5^–2^15^) and diluted serum (2^10^) were applied. Subsequently, phosphatase‐conjugated goat anti‐rat IgG (goat anti‐rat IgG/H&L antibody HRP conjugate SA00001‐15; Proteintech, Rosemont, IL, USA) was added and developed with a phosphatase substrate. The optical density at 450 nm was measured using a microplate reader, and the averages for each group were calculated.

### 2.5 Immunohistochemistry

Immediately after euthanasia, the left gastrocnemii were collected, placed in a 4% paraformaldehyde solution for fixation, and embedded in paraffin after 3 days. The paraffin‐embedded muscles were cut into 5 mm‐thick slices using a rotary microtome (MicromHM325 Rotary Microtome; Thermo Fisher Scientific, Waltham, MA, USA). The slides were activated for antigenicity using a citrate buffer solution and microwaved after paraffin removal. The slides were then immersed in a peroxidase blocking solution (BLOXALL Endogenous Peroxidase and Alkaline Phosphatase Blocking Solution; Vector Laboratories Inc., Newark, CA, USA) for 10 min. After washing with phosphate‐buffered saline (PBS), the slides were immersed in a blocking solution (2.5% normal horse serum; Vector Laboratories Inc.) for 30 min. They were then incubated overnight at 4°C with the primary antibody (mouse anti‐PGC‐1αmonoclonal antibody 66369‐1‐lg; Proteintech), which was diluted 1:100 in 2.5% normal horse serum. The sections were washed in PBS three times for 5 min each and then incubated with secondary antibodies (goat anti‐mouse IgG antibody, HRP conjugate SA00001‐1; Proteintech) for 30 min. The sections were washed three more times in PBS for 5 min each. After incubation with ABC reagent (VECTASTAIN Elite ABC Reagent; Vector Laboratories Inc.) for 30 min, the sections were incubated with diaminobenzidine for a minute. Finally, the sections were counterstained with hematoxylin and mounted using Histomount Mounting Solution (Thermo Fisher Scientific). Microphotographs of the slides were captured using a digital camera (Olympus AX70; Olympus, Tokyo, Japan) attached to a light microscope (Olympus BX51; Olympus).

### 2.6 Western Blot

Immediately after euthanasia, the right gastrocnemii were collected and stored at −80°C. The muscles were then homogenized in ice‐cold radioimmunoprecipitation assay (RIPA) buffer with protease and phosphatase inhibitors (EzRIPA Lysis kit; ATTO Corporation, Tokyo, Japan) using a polytron homogenizer (PT1200E; KINEMATICA, Lucerne, Switzerland). After removing tissue debris by centrifugation at 1000 × *g* for 15 min at 4°C, the supernatants were collected for western blotting analysis. Protein concentrations were measured using the BCA method (BCA Protein Assay Kit; Takara Bio Inc., Shiga, Japan), and adjusted to 1 mg/mL with RIPA buffer. Equal amounts of protein were separated using 10% and 15% sodium dodecyl sulfate‐polyacrylamide gel electrophoresis (SDS‐PAGE). The separated proteins were transferred onto polyvinylidene fluoride membranes. After immersion in a blocking solution (3% bovine serum albumin; FUJIFILM Wako Pure Chemical Corporation, Osaka, Japan) for 30 min, the membranes were incubated overnight at 4°C with 1:5000 (PGC‐1α), 1:10000 (TNF‐α), and 1:20000 (GAPDH) diluted primary antibodies (rabbit anti PGC‐1α polyclonal antibody PA5‐72948, Thermo Fisher Scientific; rabbit anti TNF‐α polyclonal antibody 26162‐1‐AP, Proteintech; and rabbit anti GAPDH polyclonal antibody 10494‐1‐AP, Proteintech). After washing with PBS, the membranes were incubated with secondary antibodies, diluted 1:10000 for PGC‐1α and 1:20000 for TNF‐α (goat anti‐rabbit IgG H&L antibody, horseradish peroxidase conjugate SA00001‐2; Proteintech) for 30 min. The blots were developed using Immobilon Western Chemiluminescent horseradish peroxidase (HRP) substrate (EzWestBlue W; ATTO Corporation). Detected bands were scanned using a scanner (GT‐X830; Seiko Epson Corporation, Nagano, Japan), and PGC‐α and TNF‐α expression were analyzed using image analysis software (Image J; National Institutes of Health, Bethesda, MD, USA). The measured values were averaged, and the ratios were calculated based on the average of the control group.

### 2.7 Statistical Analysis

The mean ± standard deviation (SD) of each variable was calculated, and statistical differences in body weight, serum antibody titers for IgG, PGC‐1αexpression, and TNF‐αexpression between the two groups were evaluated using the Mann–Whitney *U* test, after testing for normality using the Shapiro–Wilk test. The correlation between PGC‐1α and TNF‐α expression was analyzed using Spearman's rank correlation coefficient test. All statistical analyses were performed using statistical software (Bell Curve for Excel; Social Survey Research Information Co. Ltd., Tokyo, Japan). Statistical significance was set at *p* < 0.05.

## 3 Results

### 3.1 Body Weight

The body weight of the experimental subjects (before and after administration) as well as the weight gain are presented in Table 1. No significant difference in body weight was observed before the experiment or 3 weeks after administration of *P. gingivalis* or saline between the *P. g*‐group and the Control‐group, and there was no difference in weight gain either. No specific problems were observed in either group during the experimental period.

**Table 1:** Body weight.

	Before administration (g)	After administration (g)	Weight gain (g)
*P. g*‐group	196.13 (±22.43)	265.19 (±12.72)	69.06 (±26.76)
Control‐group	197.14 (±26.86)	271.08 (±17.64)	73.94 (±28.13)
*p*‐value	0.896	0.579	0.684

*Note:* Statistical differences in body weight between the two groups were evaluated using the Mann–Whitney *U* test, after testing for normality using the Shapiro–Wilk test. No significant difference in body weight was observed before the experiment or 3 weeks after administration of *P. gingivalis* or saline.

### 3.2 Serum Antibody Titers for IgG in Response to *Porphyromonas gingivalis*

**Figure 2:**
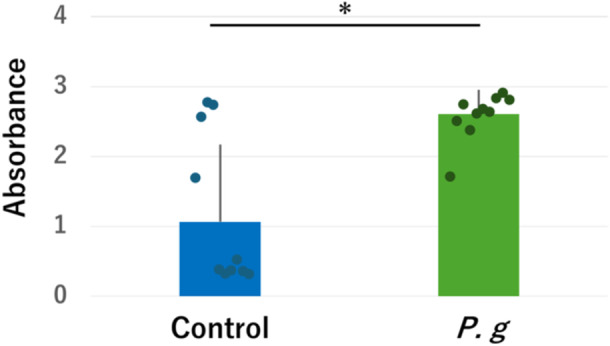
Serum antibody titers for IgG in response to *P. gingivalis*. Serum antibody titers for IgG in response to *P. gingivalis* infection were significantly higher in the *P. gingivalis* group than in the control group (*p* = 0.0015).

To evaluate the effect of *P. gingivalis* administration on the whole body, serum antibody titers for IgG were analyzed (Figure 2). *The P. g*‐group demonstrated significantly higher serum antibody titers compared to the Control‐group (*p* = 0.0015).

### 3.3 PGC‐1α Expression by Immunohistochemistry Assay

**Figure 3:**
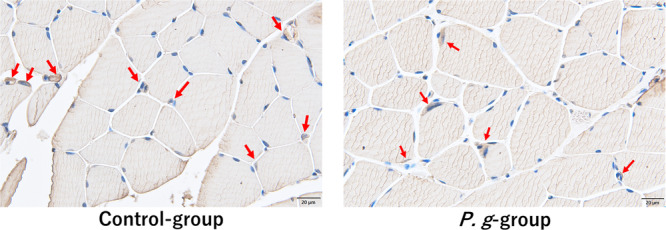
PGC‐1α expression by immunohistochemistry assay. Stained areas, indicating PGC‐1α expression, were located around the nuclei of skeletal muscle cells.

The stained area indicating PGC‐1αexpression was located around the nuclei of skeletal muscle cells. PGC‐1α expression was observed in both groups, with stained areas being more prominent in the control group than in the *P. g*‐group (Figure 3).

### 3.4 Expression of PGC‐1α and TNF‐α by Western Blot Assay

**Figure 4:**
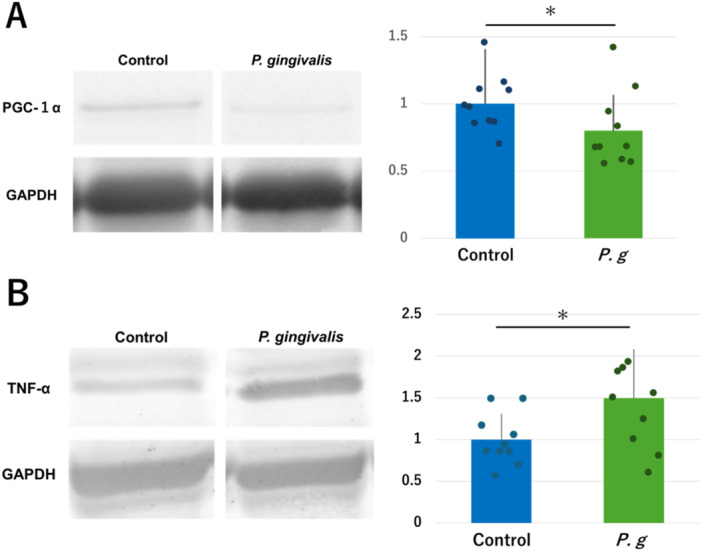
Results of Western blot analysis. The expression of PGC‐1α in skeletal muscle was significantly lower in the *P. gingivalis* group than in the control group (*p* = 0.0376, panel A). The expression of TNF‐α was significantly higher in the *P. gingivalis* group than in the control group (*p* = 0.0451, panel B).

The results of the western blot analysis are shown in Figure 4. Full‐length, uncropped western blot images are provided in Figure S1. The expression of PGC‐1α in skeletal muscle was significantly lower in the *P. g*‐group than in the Control‐group (*p* = 0.0376, Figure 4A). The expression of TNF‐α was significantly higher in the *P. g*‐group than in the Control‐group (*p* = 0.0451, Figure 4B).

**Figure 5:**
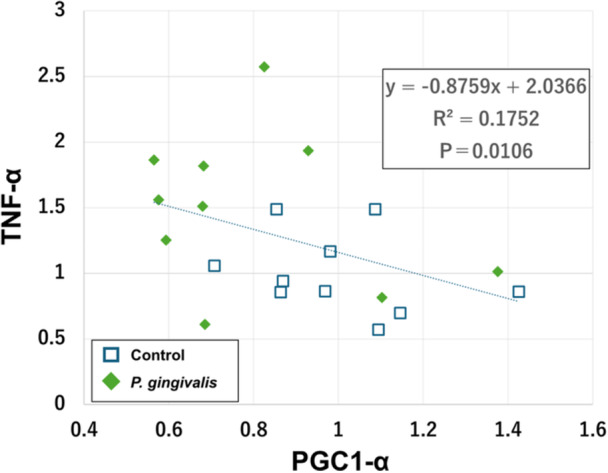
Correlation between PGC‐1α and TNF‐α expression. A significant correlation was observed between PGC‐1α and TNF‐α expression.

We also evaluate the correlation between the expression of PGC‐1α and TNF‐α, and a significant correlation was observed between the expression of PGC‐1α and TNF‐α (*p* = 0.0106, *y* = −0.8759*x* + 2.0366, *R*2 = 0.1752; Figure 5).

## 4 Discussion

In this study, we hypothesized that increasing systemic levels of *P. gingivalis* would suppress the ability to improve muscle endurance after training. Therefore, we evaluated the relationship between *P. gingivalis* infection and PGC‐1α, a key factor in mitochondrial synthesis, in vivo.

*Porphyromonas gingivalis* is a major pathogen of periodontal disease, classified within the red complex of Socransky's pathogen classification (Socransky et al. 1998). *P. gingivalis* invades blood vessels through the endothelial cells of the periodontal pocket, allowing bacterial toxins and components to access the bloodstream (Dorn et al. 2002). This contributes to the worsening of systemic syndromes such as heart disease, arteriosclerosis, and diabetes, among others (Cardoso et al. 2018).

In this study, rats in the *P. g*‐group were administered sonicated *P. gingivalis*. An increase in serum antibody titers for IgG was observed in response to *P. gingivalis* administration. This result indicates that *P. gingivalis* had spread throughout the body, as reported in previous studies on systemic effects (Kojima et al. 1997). In a previous study, we observed similar results following oral administration of sonicated *P. gingivalis*. However, due to the variations in serum antibody titers observed in the original study (Hayashi et al. 2022), we opted for intraperitoneal administration in this study.

No significant difference was observed in body weight between the two groups, and no abnormal behavior was observed in any of the rats, suggesting that *P. gingivalis* administration did not affect nutritional intake or cause any changes in the expression of PGC‐1α and TNF‐α after exercise.

After 3 weeks of administration, all rats underwent endurance training using a treadmill. The intensity used (22 m/min for 30 min) was based on previous studies that evaluated accumulated lactate levels in the blood (Farenia et al. 2019; Lesmana et al. 2016). In these studies, an intensity of 22 m/min was considered high.

Twenty‐four hours after treadmill training, skeletal muscles were extracted, and PGC‐1α expression was evaluated. In immunohistochemistry, PGC‐1α expression was observed around the nuclei of skeletal muscle. PGC‐1α expression levels begin to increase a few hours after training, leading to an increase in mitochondria within a few days (McGee and Hargreaves 2020). To minimize the influence of the time elapsed since training, all samples were collected 24 h post‐training.

PGC‐1α expression was significantly lower in the rats of the *P. g*‐group than in the Control‐group, suggesting that *P. gingivalis* infection suppresses PGC‐1α expression. We also evaluated the expression of TNF‐α (an inflammatory cytokine) in skeletal muscle and found increased TNF‐α expression in the *P. gingivalis*‐administered rats. This increase may be explained by the binding of *P. gingivalis* antigens to Toll‐like receptors (TLRs) on macrophages and dendritic cells, resulting in the nuclear translocation of NF‐κB and subsequent upregulation of pro‐inflammatory cytokines such as TNF‐α (Zhou et al. 2005; Burns et al. 2006; Hajishengallis et al. 2009). Alternatively, stimulation of macrophages by gingipains may also have contributed to the increased TNF‐α expression (Grenier and Tanabe 2010). Additionally, a negative correlation between PGC‐1α and TNF‐α expression was observed. These results suggest that administration of *P. gingivalis* increased TNF‐α, leading to suppressed PGC‐1α expression in skeletal muscle after exercise. It is likely that TNF‐α bound to receptors such as TNFR1, leading to IKK activation, IκB degradation, and subsequent NF‐κB activation, which in turn suppresses PGC‐1α gene expression (Palomer et al. 2009). This finding has been previously reported: A previous study showed that increased TNF‐α suppresses PGC‐1α expression (Palomer et al. 2009), suggesting that *P. gingivalis* infection induces TNF‐α expression in skeletal muscle, thereby affecting PGC‐1αexpression post endurance training.

The authors acknowledge several limitations in the present study. We only evaluated the effect of *P. gingivalis* despite the presence of other periodontal disease pathogens, such as *Treponema denticola* and *Tannerella forsythia*, which belong to the red complex according to Socransky's pathogen classification (Holt and Ebersole 2005). Nevertheless, *P. gingivalis* is considered one of the most important periodontal pathogens associated with periodontitis. Previous studies suggest that *P. gingivalis* does not independently induce periodontitis, but rather acts as a keystone pathogen that promotes disease progression by altering the oral microbiome (Hajishengallis et al. 2012). However, an increased abundance of *P. gingivalis* is consistently observed in patients with periodontitis, and *P. gingivalis* itself reportedly exerts detrimental systemic effects, including the induction of inflammatory responses in distant tissues (Olsen and Yilmaz 2016). Therefore, investigating the effects of *P. gingivalis* alone is scientifically meaningful. Additionally, changes in the oral biome were reported to affect periodontal disease. Therefore, evaluating the effects of other pathogens on the relationship between periodontitis and muscle endurance is necessary and should be explored in future research. Furthermore, we evaluated PGC1‐αexpression but did not directly measure mitochondrial synthesis or ATP production. The activation of either mitochondrial transcription factor A (TFAM) or cytochrome c oxidase subunit 4 (COXIV)/succinate dehydrogenase complex flavoprotein subunit A (SDHA) on the mitochondrial side, which constitutes the functional consequences of PGC‐1α expression, should also be determined to demonstrate the occurrence of metabolic changes. Further studies should measure these factors for a comprehensive evaluation of muscle endurance.

The peritoneal injection of sonicated *P. gingivalis* caused infectious and inflammatory conditions, evidenced by increased production of IgG against *P. gingivalis* and TNF‐α. These findings suggest an elevated immune response and ongoing inflammation. However, the inflammatory profile (IL‐6, IL‐1β, and acute phase response) as well as muscle‐specific inflammation were not evaluated as part of this study, which constitutes one of its limitations. Evaluating the involvement of p‐NF‐κB p65 or IκBα may also be useful to better interpret the obtained results.

Furthermore, activity levels were not evaluated. Total running distance should be evaluated in future experiments, as well as the levels of proteins stimulated by exercise, such as p‐AMPK and p‐p38. In addition, data on baseline levels of PGC‐1α before exercise were not collected, which prevented an evaluation of changes in these levels after treadmill training. However, an increase in PGC‐1α levels after endurance training has been previously reported (Olesen et al. 2010). Baseline levels before exercise should be evaluated in future studies.

Finally, relatively young rats (aged 8–11 weeks) were used in this study. The age range of the experimental subjects should be expanded in future studies, which would allow for the evaluation of a wider range of potential effects that may only become evident in older rats.

Despite these limitations, this study represents the first step towards exploring the relationship between periodontal pathogens and skeletal muscle endurance.

## 5 Conclusion

In this study, rats administered *P. gingivalis* demonstrated suppressed PGC‐1α expression after endurance training. This suggests that *P. gingivalis* infection, originating from the oral cavity and invading the bloodstream via the periodontal pocket, may impair the acquisition of endurance after training. Understanding the effect of *P. gingivalis* infection on muscle endurance is important for improving the well‐being of both healthy individuals and those with periodontitis.

## Author Contributions

**Kairi Hayashi:** conceptualization, methodology, investigation, formal analysis, data curation, visualization, funding acquisition, writing – original draft. **Yasuo Takeuchi:** conceptualization, methodology, writing – review and editing. **Hiroaki Kobayashi:** conceptualization, methodology, writing – review and editing. **Gen Tanabe:** investigation, writing – review and editing. **Hiroshi Churei:** investigation, writing – review and editing. **Toshiaki Ueno:** supervision, writing – review and editing. **Kenji Fueki:** supervision, writing – review and editing.

## Ethics Statement

All animal experiments were approved by the Institutional Animal Care and Use Committee of the Institute of Science, Tokyo (A2022‐094C2).

## Consent

Patient consent was not required for this study as it did not involve human participants.

## Conflicts of Interest

The authors declare no conflicts of interest.

## Supporting information


Supporting File

## Data Availability

The data are available from the corresponding author on reasonable request.
