# Supplementary material for: Effect of Porphyromonas gingivalis Infection on PGC‐1α in Skeletal Muscle After Endurance Training In Vivo
Source: Clin Exp Dent Res. 2026 Jul 16;12(4):e70415. doi: 10.1002/cre2.70415 (PMC13375082; doi:10.1002/cre2.70415)

Supplementary Material

**Full-length Western blot images: PGC-1α**

The expression of PGC-1α in skeletal muscle was significantly lower in the P. g-group than in the Control-group.


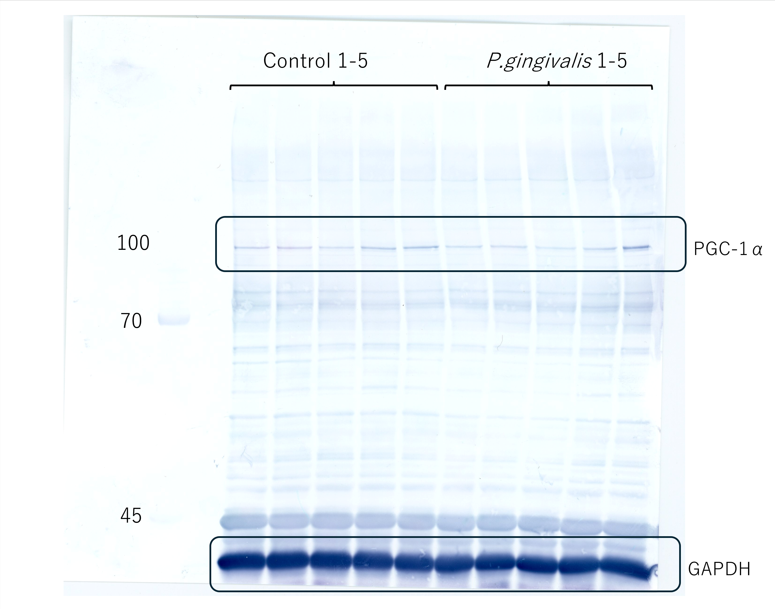


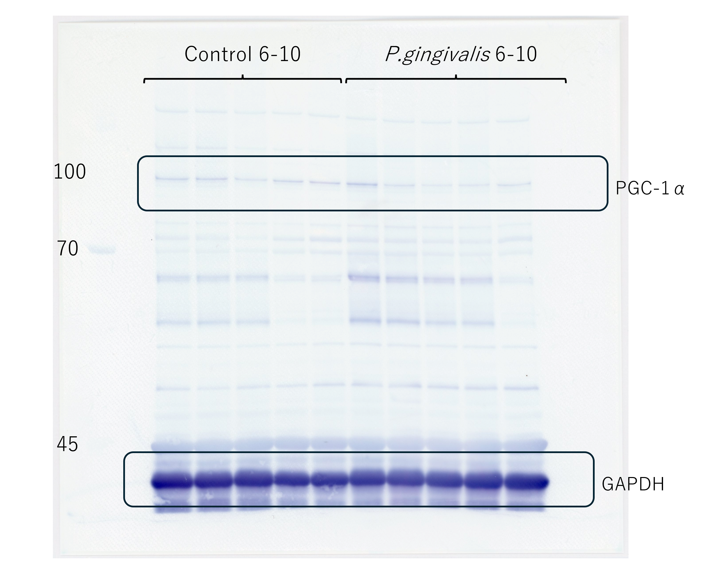


**Full-length Western blot images: TNF-α**

The expression of TNF-α was significantly higher in the P. g-group than in the Control-group.


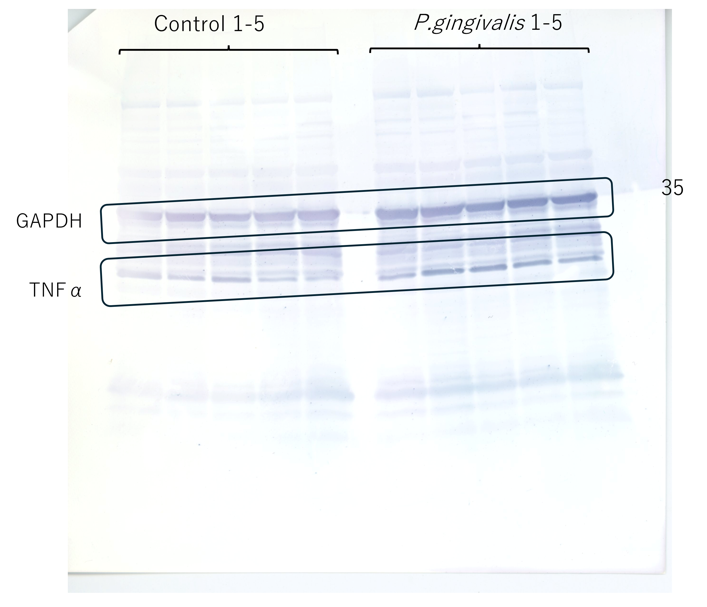


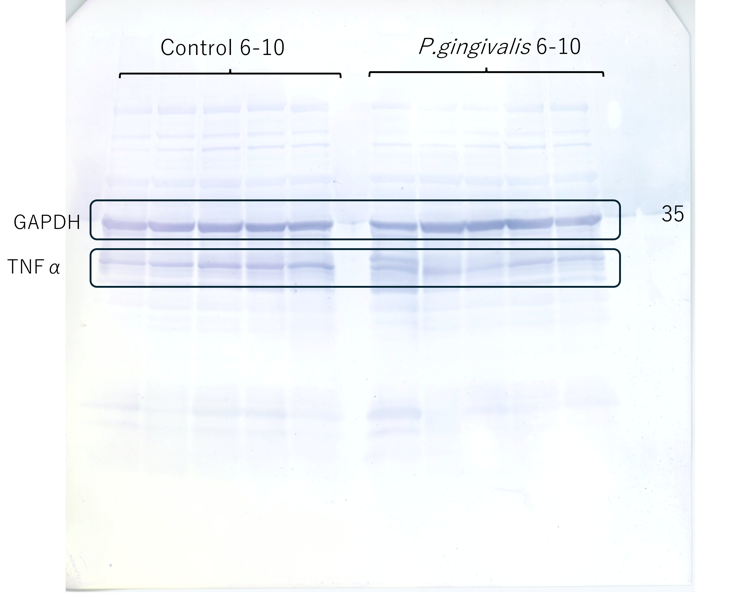

Supplement: Supplementary file 1 — Supporting File [file CRE2-12-e70415-s001.docx]
